# Dual inhibition of the *Echinococcus multilocularis* energy metabolism

**DOI:** 10.3389/fvets.2022.981664

**Published:** 2022-08-05

**Authors:** Sheena Chaudhry, Raphael Zurbriggen, Matías Preza, Tobias Kämpfer, Marc Kaethner, Roman Memedovski, Nathalie Scorrano, Andrew Hemphill, Joseph Stone Doggett, Britta Lundström-Stadelmann

**Affiliations:** ^1^Institute of Parasitology, Vetsuisse Faculty, University of Bern, Bern, Switzerland; ^2^Graduate School for Cellular and Biomedical Sciences, University of Bern, Bern, Switzerland; ^3^Department of Infectious Diseases and Pathobiology, Oregon Health and Science University, Portland, OR, United States; ^4^Department of Infectious Diseases and Pathobiology, Veterans Affairs Portland Health Care System, Portland, OR, United States

**Keywords:** mitochondrium, ELQ, endochin-like quinolone, malate dismutation, cytochrome *bc*_1_, electron transfer chain, drug repurposing

## Abstract

Alveolar echinococcosis is caused by the metacestode stage of the zoonotic parasite *Echinococcus multilocularis*. Current chemotherapeutic treatment options rely on benzimidazoles, which have limited curative capabilities and can cause severe side effects. Thus, novel treatment options are urgently needed. In search for novel targetable pathways we focused on the mitochondrial energy metabolism of *E. multilocularis*. The parasite relies hereby on two pathways: The classical oxidative phosphorylation including the electron transfer chain (ETC), and the anaerobic malate dismutation (MD). We screened 13 endochin-like quinolones (ELQs) *in vitro* for their activities against two isolates of *E. multilocularis* metacestodes and isolated germinal layer cells by the phosphoglucose isomerase (PGI) assay and the CellTiter Glo assay. For the five most active ELQs (ELQ-121, ELQ-136, ELQ-271, ELQ-400, and ELQ-437), EC_50_ values against metacestodes were assessed by PGI assay, and IC_50_ values against mammalian cells were measured by Alamar Blue assay. Further, the gene sequence of the proposed target, the mitochondrial cytochrome *b*, was analyzed. This allowed for a limited structure activity relationship study of ELQs against *E. multilocularis*, including analyses of the inhibition of the two functional sites of the cytochrome *b*. By applying the Seahorse XFp Extracellular Flux Analyzer, oxygen consumption assays showed that ELQ-400 inhibits the *E. multilocularis* cytochrome *bc*_1_ complex under normoxic conditions. When tested under anaerobic conditions, ELQ-400 was hardly active against *E. multilocularis* metacestodes. These results were confirmed by transmission electron microscopy. ELQ-400 treatment increased levels of parasite-released succinate, the final electron acceptor of the MD. This suggests that the parasite switched to MD for energy generation. Therefore, MD was inhibited with quinazoline, which did not induce damage to metacestodes under anaerobic conditions. However, it reduced the production of succinate compared to control treated parasites (i.e., inhibited the MD). The combination treatment with quinazoline strongly improved the activity of the *bc*_1_ inhibitor ELQ-400 against *E. multilocularis* metacestodes under anaerobic conditions. We conclude that simultaneous targeting of the ETC and the MD of *E. multilocularis* is a possible novel treatment approach for alveolar echinococcosis, and possibly also other foodborne diseases inflicted by platyhelminths, which cause substantial economic losses in livestock industry.

## Introduction

Alveolar Echinococcosis (AE) is a lethal disease caused by infections with the fox tapeworm *Echinococcus multilocularis*, which is endemic to the Northern hemisphere. AE is the highest ranked food-borne parasitic disease in Europe (1) causing at least 18'500 new human cases of AE each year and more than 666,400 disability-adjusted life years (DALYs) with case numbers rising (2). AE is a neglected and emerging disease and until now, AE is an uncontrolled health problem of particular concern in developing and resource-poor regions (3).

The natural life cycle of *E. multilocularis* includes foxes and other canids as definitive hosts. In these definite hosts adult tapeworms grow in the intestines and release parasite eggs into the environment *via* the host's feces. Intermediate hosts, such as small rodents, are infected upon accidental oral uptake of these eggs. Also humans and other accidental hosts such as monkeys, pigs and dogs, can get infected by the parasite *via* oral uptake of eggs. This may lead to the establishment of a multivesicular larval stage, the metacestode, which grows mainly in the liver, thereby causing the disease AE (4). Metacestodes are surrounded by a protective carbohydrate-rich laminated layer (LL). Adjacent to the LL, the germinal layer (GL) is formed, which consists of muscle cells, nerve cells, glycogen storage cells, connective tissue sub-tegumentary cytons, and undifferentiated stem cells (5). Eventually, metacestodes form brood capsules and protoscoleces which, when taken up by a suitable final host, will grow into adult tapeworms in the final host's intestines.

AE is a chronic disease characterized by a tumor-like growth of the metacestodes with the potential for metastasis formation (3). In the progressive stage of AE, non-specific symptoms such as abdominal pain, jaundice, cholestasis, hepatomegaly, fever, anemia, weight loss, and pleural pain appear (3). If untreated, the infection will reach a final advanced stage, during which severe hepatic dysfunction occurs. If not treated properly, or if treatment fails, the infection leads to death due to malfunction of the liver or other affected organs (6).

Surgical removal is the only curative option for treatment of AE, but it cannot always be applied. Drug treatment is based on albendazole or mebendazole, but these drugs are not always effective, often act parasitostatically, and they have to be taken daily and life-long. Although benzimidazole treatment has significantly increased the 10-year survival rate for patients, it poses a considerable burden to patients, also, because side-effects frequently occur (7). All these shortcomings make it urgent to develop alternative chemotherapeutic options against AE (6).

As AE is a neglected disease, development of treatment options receives hardly any financial or industrial attention. Therefore, drug repurposing is a valuable method, which can allow for the identification of potential new drugs by use of already known drugs and drug-classes from other fields of disease (8). The advancements in the *in vitro* culture of *E. multilocularis* metacestodes, as well as the establishment of reliable *in vitro* drug testing systems, allow for screening and characterization of repurposed drugs against AE (6). This makes *E. multilocularis* an excellent model to study basic concepts of drug-treatment against liver-infecting platyhelminths *in vitro*. In the future, findings could be transferred to related parasites, such as *E. granulosus, Fasciola* spp., and others, which cause substantial economic losses in livestock industries (9, 10).

The energy metabolism of *E. multilocularis* might provide a valuable future target, because the generation of energy is central to any organism. Like any eukaryotic life form, also *E. multilocularis* uses glucose as a prime energy source, and metabolizes it through glycolysis and, to a small extent, fermentation, as well as through mitochondrial pathways. Interestingly, *E. multilocularis* applies two mitochondrial pathways for energy generation: (i) the classical electron transfer chain (ETC) coupled to oxidative phosphorylation and (ii) the malate dismutation (MD) (11). In the classical pathway pyruvate produced through glycolysis is imported into the mitochondria and metabolized through the citric acid cycle. Further, electrons are passed through the ETC consisting of complexes I-IV and this finally leads to the generation of ATP, which is depending on oxygen as an electron acceptor. Thus, this classical pathway can only function in the presence of oxygen. The second pathway, the MD, is an oxygen-independent pathway found in all helminths, marine invertebrates and euglenids, *Trypanosoma* spp. and bacteria (12–15), but it is absent from mammals. This difference in energy metabolism compared to mammalian host species renders the MD an interesting drug target.

MD includes a partial inversion of the citric acid cycle with complex II working as a fumarate reductase rather than a succinate dehydrogenase, and instead of ubiquinone, rhodoquinone is used to transfer electrons between complexes I and II. Complex I hereby translocates protons across the inner mitochondrial membrane, which finally leads to the generation of ATP by ATP synthase even in the absence of oxygen. Succinate is released as a final electron acceptor. In addition, MD also includes the oxidation of malate to pyruvate and acetate. Thus, the final electron acceptors of MD are succinate in one branch, and acetate in the other branch, and these products are strongly released by many helminths, also *E. multilocularis* (11, 15).

Few studies have tackled the energy metabolism of *E. multilocularis* in the past: Enkai *et al*. showed that a combined inhibition of mitochondrial complexes II and III by atpenin A5 and atovaquone, respectively, killed protoscoleces *in vitro* (16). It was also shown that buparvaquone efficiently inhibited complex III (also called cytochrome *bc*_1_ complex) and it was effective against metacestodes *in vitro* (17). The same study also identified the endochin-like quinolone (ELQ) ELQ-400 as highly active against *E. multilocularis* metacestodes *in vitro*, but the compound was not further characterized at that time (17). ELQs are potent inhibitors of the cytochrome *bc*_1_ complex of a variety of apicomplexan parasites like *Plasmodium falciparum* (18, 19), *Toxoplasma gondii* (20–22)*, Neospora caninum* (23, 24), *Babesia* spp. and *Theileria equi* (25–27), and *Besnoitia besnoiti* (28). One study examined the MD of *E. multilocularis* and showed that quinazolines can inhibit the complex I, and thereby the MD, of protoscoleces (29).

As these above-mentioned studies pointed toward the big potential of inhibitors of the mitochondrial energy pathways of *Echinococcus*, the present paper laid further focus on the comparative testing of a series of ELQs against two different isolates of *E. multilocularis*, and a combined inhibition of the classical ETC and the MD, to treat *E. multilocularis* metacestodes.

## Materials and methods

**Figure 1:**
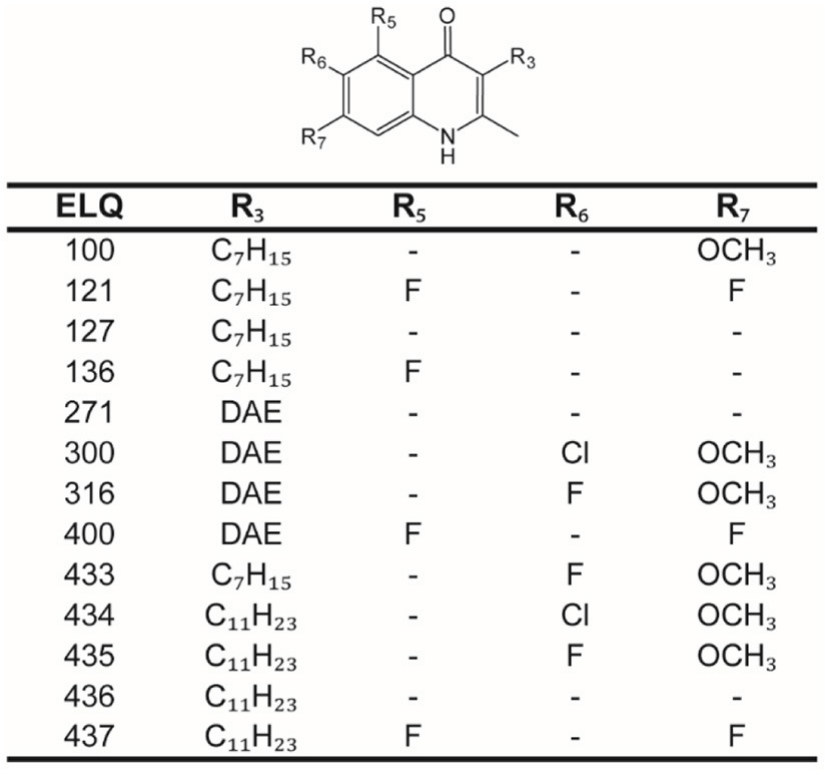
Structures of endochin-like quinolones (ELQs). Core structure of ELQs with numbered residues for each of the here tested ELQs. ELQ numbers are given in the first column, and the substituents (R3, R5, R6, and R7) are given in the following columns. DAE, diaryl ether.

All chemicals were purchased from Sigma-Aldrich (St. Louis, Mo, USA), if not stated otherwise. Dulbeccos's modified Eagle medium (DMEM) and fetal bovine serum (FBS) were purchased from Biochrom GmbH (Berlin, Germany). Trypsin/ EDTA (Trypsin 0.05%/ EDTA 0.02%) was bought from LuBioScience GmbH (Luzern, Switzerland). Penicillin and Streptomycin (10'000 Units/mL Penicillin, 10'000 μg/mL Streptomycin) was supplied by Gibco (Fisher Scientific AG, Reinach, Switzerland). The 13 ELQs (ELQ-100, ELQ-121, ELQ-127, ELQ-136, ELQ-271, ELQ-300, ELQ-316, ELQ-400, ELQ-433, ELQ-434, ELQ-435, ELQ-436, and ELQ-437) were synthetized as described before (22) and prepared as 10 mM solutions in DMSO, stored as aliquots at minus 20°C. Before use, compounds were shaken for 10 min at 45°C and vortexed well. The structures of ELQs were prepared in ACD/ChemSketch (ACD Labs, Toronto, Canada, Version 2020.2.1) and they are given in Figure 1.

### Mice and ethics statement

For parasite maintenance animals were purchased from Charles River Laboratories (Sulzheim, Germany) and used for experimentation after 2 weeks of acclimatization. Female BALB/c mice were maintained in a 12 h light/dark cycle, controlled temperature of 21–23°C, and a relative humidity of 45–55%. Food and water was provided *ad libitum*. All animals were treated in compliance with the Swiss Federal Protection of Animals Act (TSchV, SR455), and experiments were approved by the Animal Welfare Committee of the canton of Bern under the license number BE30/19.

### *In vitro* culture of *E. multilocularis* metacestodes

*E. multilocularis* metacestodes were maintained as previously described by Rufener *et al*. (17). Metacestodes [isolates H95 (30) and Sval (31)] were grown in intraperitoneally infected mice and parasite material was resected after 4 months of parasite growth. *In vitro* cultures were initiated and cultured as described by Rufener *et al*. (17) in DMEM containing 10% FBS, 100 U/mL penicillin, 100 μg/mL streptomycin and 10 μg/mL tetracycline, and in co-culture with Reuber rat hepatoma (Rh) cells at 37°C with 5% CO_2_ under humid atmosphere.

### *In vitro* drug testing against *E. multilocularis* metacestodes

To assess the *in vitro* efficacy of ELQs against *E. multilocularis*, (a) the damage-marker phosphoglucose isomerase (PGI) assay was applied against metacestodes, and (b) the germinal layer (GL) cell viability assay was applied to measure effects on isolated GL cells. Both assays are described in more detail below. The drug testing strategy was as follows: PGI assay was first conducted for overview screening at 10 μM with the parasite isolates H95 and Sval. The GL cell viability assay was performed at 0.1 μM with both isolates H95 and Sval. For active compounds (PGI > 20% activity compared to Tx-100 positive control; GL cell viability < 30% of DMSO negative control), PGI assay was applied for EC_50_ measurements based on the H95 isolate.

#### *In vitro* testing of ELQs against *E. multilocularis* metacestodes by PGI assay

The PGI assay measures the amount of PGI that metacestodes release into the medium when damaged (17, 32). The purification and *in vitro* drug incubation of *E. multilocularis* metacestodes was conducted according to Stadelmann *et al*. (32). In short, cultures were applied for *in vitro* drug testing at the age of 6-8 weeks. *E. multilocularis* metacestodes were therefore purified by the addition of 2% saccharose and washed extensively with PBS. Purified metacestodes were suspended 1:3 with DMEM (without phenol red, supplemented with 100 U/mL penicillin, 100 μg/mL streptomycin) and distributed in 48-well plates (1 mL/well, thus 300 μL pure metacestode vesicles per well). ELQs were added to the wells to the desired concentrations (10 μM for screening, 30–0.12 μM in 1:2 dilution steps for EC_50_ measurements) in 0.1% DMSO. The corresponding DMSO concentration was used as negative control and Tx-100 (0.1% final concentration) was used for total damage (positive control). The plates were incubated for 5 days under normoxic conditions (37°C; 5% CO_2_, 21% O_2_, humid atmosphere). After 5 days, relative PGI activities were measured and calculated as percentage of the Tx-100 control as described previously in Rufener *et al*. (17). They were calculated as mean values and standard deviations for each triplicate. EC_50_ calculations were calculated based on logit-log transformed values in Microsoft Office Excel 2010. EC_50_ measurements were performed twice, and averages and standard deviations of these repeated tests are given.

#### *In vitro* testing of ELQs against *E. multilocularis* GL cells by viability assay

The isolation of *E. multilocularis* GL cells was done as described previously (17) with minor modifications. Fifteen units of GL cells were distributed to each well of a 384 well plate in a total volume of 12.5 μL conditioned medium. The different ELQs were added to the wells to 0.1 μM, in quadruplicates. As a negative control 0.1% DMSO was used. The 384-well plate was incubated for 5 days at 37°C under humid nitrogen atmosphere. Thereafter, viability of the GL cells under different drug treatments was assessed by CellTiter-Glo® (Promega AG, Dübendorf, Switzerland). The CellTiter-Glo reagent was complemented with 1% Tx-100. The luciferase bioluminescence was measured at 530 nm by using the Enspire multilabel reader (PerkinElmer Life Sciences, Schwerzenbach, Switzerland). Using Microsoft Excel, the blank values were subtracted from the experimental values and the relative viability compared to DMSO was calculated. Viabilities were calculated as mean values and standard deviations for each quadruplicate. Calculations were made based on logit-log transformed values in Microsoft Office Excel 2010.

### Mammalian cell toxicity assay

For the compounds with activity against *E. multilocularis* (ELQ-121, ELQ-136, ELQ-271, ELQ-400, and ELQ-437), the toxicity against mammalian cells was assessed according to Rufener *et al*. (17) by Alamar Blue assay on Rh cells, at confluent and pre-confluent state. Compounds were added in a serial dilution (30–0.23 μM, 1:2 dilution steps) and in triplicates. Plates were incubated at 37°C, 5% CO_2_ and humid atmosphere for 5 days. Resazurin was added to a final concentration of 10 mg/L to each well, and fluorescence measured at 530 nm on an Enspire multilabel plate reader over 3 h. By using Microsoft Office Excel 2010 and a logit-log transformation of the relative growth, IC_50_ values were calculated. The test was performed twice for each compound, and respective averages and standard deviations are given.

### Transmission electron microscopy (TEM)

*E. multilocularis* metacestodes treated with ELQ-400 (0.2, 1 and 2 μM, 5 days of incubation) under normoxic (37°C, 21% O_2_, 5% CO_2_, humid atmosphere) and anaerobic conditions (37°C, 80% N_2_, 10% CO_2_, and 10% H_2_, humid atmosphere) were processed for TEM as previously described (17). Metacestodes were washed in 100 mM sodium cacodylate buffer (pH 7.3), and were fixed in 2% glutaraldehyde in cacodylate buffer overnight at 4°C. Following several washes in cacodylate buffer, metacestodes were post-fixed in 2% osmium tetroxide in cacodylate buffer for 3–4 h, washed in water, and samples were treated with Uranyless™ solution (EMS, Hatfield, PA, USA) for 20 min, followed by several washes in distilled water. Specimens were dehydrated in a graded series of ethanol and were embedded in EPON-812 epoxy resin. Polymerization of the resin was carried out at 60°C for 24 h. Ultrathin sections were cut on a Reichert and Jung (Vienna) microtome, and were allowed to settle onto 400 mesh formvar carbon coated grids (Plano GmbH, Wetzlar, Germany). Specimens were contrasted with Uranyless and lead citrate, and were viewed on a FEI Morgagni Transmission Electron Microscope operating at 80 kV.

### Seahorse assay

To measure effects of ELQ-400 on the oxygen consumption rate (OCR), Seahorse assay was conducted according to the method established by Rufener *et al*. (17) and according to the manual for Seahorse XFp Extracellular Flux Analyzer (Agilent Technologies, Bucher Biotec, Basel, Switzerland). *E. multilocularis* GL cells were isolated and prepared the day before. Cells were centrifuged and resuspended in Mitochondrial Assay Solution (MAS) solution (220 mM mannitol, 70 mM sucrose, 10 mM KH_2_PO_4_, 5 mM MgCl_2_, 2 mM HEPES, and 1 mM EGTA pH 7.2). This cell suspension was centrifuged again and the pellet was resuspended in assay-buffer (MAS, 10 mM succinate, 2 μM rotenone, 4 mM ADP, and 3.6 nM PMP). 100 units of GL cells were distributed to each well of a Seahorse XFp miniplate (Agilent Technologies) pre-coated with CellTak according to the instruction manual (Fisher Scientific, Schwerte, Germany). Two wells were used as blanks with assay buffer without cells. ELQ-400 (1 μM) was diluted in MAS solution and loaded to the delivery ports of the sensor cartridge of the first injection (or DMSO as a control). In the second injection, succinate (10 mM), glycerol 3-phosphate (10 mM), or ascorbate (20 mM) with TMPD (0.6 mM) was added. Subsequently, the measurement on the Seahorse machine (Agilent Technologies) was started with three measurements for the base line, four after the first injection, and 10 after the second injection. For each measurement 30 s mix, 30 s delay, and 2 min measure was applied. Each drug setup was tested in triplicates and the read-out and analysis was done using the software Wave (Agilent Technologies, Bucher Biotec, Basel, Switzerland). The test was repeated two times independently.

### Sequence comparisons

Sequences of cytochrome *b* were obtained from Uniprot (33) for *E. multilocularis* (C6L2E3), *T. gondii* (S8EQL3) and *P. falciparum* (Q02768) and *H. sapiens* (P00156), and from WormBase ParaSite (34) version 16 for *E. granulosus* (EgrG_900000100). Alignments were performed with Clustal (35), using the program Bioedit (36). The Qo and Qi sites of the complex were identified based on previous literature (22).

### *In vitro* drug testing against *E. multilocularis* metacestodes by combination treatments

For double inhibition of the mitochondrial energy pathways of *E. multilocularis*, quinazoline as an inhibitor of the anaerobic MD (29) was applied in combination with ELQ-400 as an inhibitor of the ETC. Both compounds were tested either alone or in combination at 10 μM with the above-described PGI assay for 5 days for their activity against *E. multilocularis* metacestodes. PGI assays were done in triplicates and two times independently. Drug incubations were performed at three different conditions for the PGI assay: normoxic (37°C, 21% O_2_, 5% CO_2_, humid atmosphere) and anaerobic conditions (37°C, 80% N_2_, 10% CO_2_, and 10% H_2_, humid atmosphere). Results of the assays were calculated as mean values and standard deviations for each biological triplicate were calculated as relative percentage to the Tx-100 control. To assess direct effects on the MD, succinate levels were measured in the supernatants of the PGI assay, as described below. To determine whether differences between groups were significant, data was subjected to student's *t*-test analysis and *P*-values below 0.05 are given.

### Assessment of succinate concentrations in culture supernatants

Succinate measurements in supernatants of drug-treated *E. multilocularis* metacestodes was performed with the “Succinic Acid Assay Kit” (Megazyme, Bray, Ireland). For the standard curve, a serial dilution (1:2) from 0.125 to 4 μg succinate per well was prepared. 20 μL of each diluted (1:20) sample or standard was used in triplicate for succinate measurements in 96-well plates (flat bottom, Greiner Bio-One GmbH, Frickenhausen, Germany). The reaction mix buffer was prepared as described in the supplier manual: per well 180 μL H_2_O, 20 μL solvent 1 (buffer, pH 8.4, sodium azide 0.02% w/v), 20 μL solvent 2 (NADH plus stabilizer), 20 μL solvent 3 (ATP, PEP, and CoA) and 2 μL solvent 4 (pyruvate kinase plus L-lactate dehydrogenase suspension) were added. After a 180 s pre-incubation time, a first measurement at 340 nm was done on an EnSpire multilabel plate reader (PerkinElmer Life Sciences, Schwerzenbach, Switzerland). 10 μL solvent 5 (succinyl-CoA synthetase suspension; 1:10 diluted) were then added to each well and the plate was read at 340 nm for 1 h every minute. Calculation of succinate concentrations was done by regression analysis using Microsoft Office Excel 2010. Succinate levels are shown as mean values with standard deviations of biological triplicates. To determine whether differences between groups were significant, data was subjected to student's *t*-test analysis and *P*-values below 0.05 are given.

## Results

### ELQs show activity against *E. multilocularis* metacestodes and GL cells

Results of the overview screening of 13 different ELQs against *E. multilocularis* isolates H95 and Sval is shown in Table 1. Tested against the Sval isolate, the PGI assay, which measures the overall damage to metacestodes, showed an activity for compounds ELQ-121, ELQ-271, ELQ-400, and ELQ-437 at 10 μM. Against the H95 isolate, ELQ-136, ELQ-271, ELQ-316, ELQ-400, and ELQ-437 were active. In the GL cell viability assay, which measures the activity of compounds against the viability of isolated GL cells of *E. multilocularis*, the compounds ELQ-121, ELQ-136, ELQ-271, ELQ-400, ELQ-436, and ELQ-437 were active against the Sval isolate. For the H95 isolate, the same compounds were active, and in addition also ELQ-127, ELQ-435, and ELQ-436.

**Table 1:** *In vitro* efficacy of ELQs against metacestodes and GL cells of two isolates of *E. multilocularis*.

**ELQ**	**Isolate Sval**		**Isolate H95**	
	**Metacestodes**	**GL cells**	**Metacestodes**	**GL cells**
100	0 (0.4)	57.9 (5.7)	1.4 (0.1)	100.1 (8.3)
121	20.9 (15.3)	7.3 (3.8)	15.2 (4.0)	1.0 (0.3)
127	2.5 (0.8)	40.4 (2.7)	11.7 (0.2)	17.2 (9.5)
136	7 (7.6)	15.1 (4.7)	21.4 (2.2)	8.4 (11.7)
271	56.9 (4.3)	25.4 (7.2)	33 (1.7)	2.7 (1.6)
300	7.6 (1.1)	46.9 (9.6)	6.6 (2.2)	84.0 (7.0)
316	4.6 (1.3)	58 (5.3)	21.4 (0.1)	81.1 (9.5)
400	27 (10.5)	14.6 (8.6)	21.4 (2.5)	8.4 (4.8)
433	1.8 (1.6)	62.1 (6.3)	4.3 (4.2)	96.4 (17.7)
434	0.6 (1.1)	81.3 (5.8)	0.4 (0.4)	33.3 (6.4)
435	0 (0.9)	80.1 (13.1)	2.6 (1.6)	15.6 (14.0)
436	3.1 (2.6)	16.4 (1.8)	3.4 (1.2)	14.0 (9.7)
437	33.1 (7.2)	19.1 (5.7)	37.6 (0.5)	19.7 (13.3)

*ELQ numbers are given in the first column. The two parasite isolates Sval and H95 were tested. Efficacy against metacestodes was assessed by PGI assay (relative to the positive control Tx-100), and against GL cells by viability assessment via ATP measurements (relative to the DMSO control) in μM. Compounds were considered to be active, if they reached a threshold of >20% against metacestodes, and <30% against GL cells. This overview screen was performed once in triplicates, and respective mean relative values are given with standard deviations in parentheses*.

Thus, overall, several compounds were active against both parasite isolates, and in both assays. Compounds that showed activity in three out of four assays were selected as positive hits and further characterized. These are: ELQ-121, ELQ-136, ELQ-271, ELQ-400, and ELQ-437. EC_50_s were calculated for metacestodes, IC_50_ies for the cytotoxicity assessed on confluent and pre-confluent Rh cell lines. Results are given in Table 2. All five ELQs showed an EC_50_ of around 0.2–2 μM against intact metacestodes (Table 2). None of the compounds was toxic on confluent Rh cells within the tested range of concentrations, whereas ELQ-271 and ELQ-400 exhibited some toxicity on pre-confluent Rh cells (8.64 and 1.46 μM, respectively). ELQ-400 was chosen for further characterization, as it was the most active compound that was previously characterized against *E. multilocularis* and applied in *in vivo* studies in mice (22), albeit other compounds tested here promised a potentially better therapeutic window based on *in vitro* data.

**Table 2:** Halfmaximal activities of ELQs against *E. multilocularis* metacestodes and Reuber rat hepatoma cells.

**ELQ**	**EC_50_ metacestodes**	**IC_50_ Rh preconfluent**	**IC_50_ Rh confluent**
121	1.19 (0.52)	>30	>30
136	0.22 (0.03)	>30	>30
271	0.24 (0.13)	8.64 (1.05)	>30
400	1.06 (0.60)	1.46 (0.17)	>30
437	1.71 (0.01)	>30	>30

*The first number indicates the ELQ numbers. Efficacy against metacestodes (isolate H95) was assessed by PGI assay (relative to the positive control Tx-100) and EC_50_ values are given in μM (concentration range tested: 30–0.12 μM). Activity against mammalian cells (Reuber rat hepatoma cells, Rh) was assessed by Alamar blue assay (relative to the DMSO control), and IC_50_ values are given for preconfluent and confluent cells is given in μM (range tested: 30–0.23 μM). All tests were performed twice with triplicates for each concentration, and respective mean relative values are given with standard deviations in parentheses*.

### Morphological effects induced by ELQ-400 on *E. multilocularis* metacestodes

**Figure 2:**
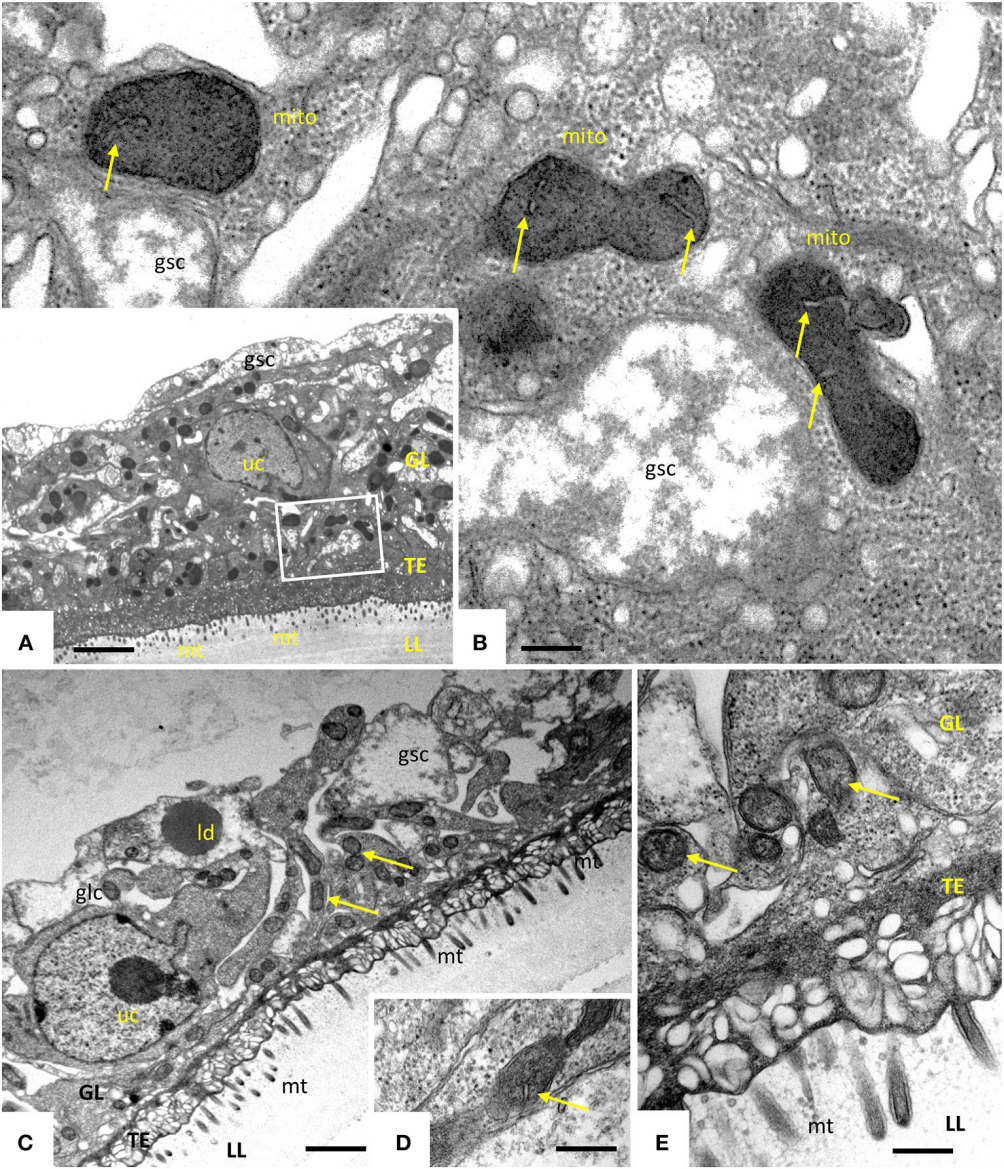
Transmission electron microscopy (TEM) of *E. multilocularis* metacestodes treated with 0.2 μM ELQ-400. TEM of non-treated metacestodes **(A,B)** and metacestodes treated with 0.2 μM ELQ-400 **(C,D)** under normoxic (21% O_2_) conditions. The boxed area in **(A)** is shown at higher magnification in **(B)**. Parasites are comprised of an outer laminated layer (LL), a tegument (TE) with numerous microtriches (mt) protruding into the LL, and the GL comprised of various cell types, including glycogen storage cells (gsc) and undifferentiated cells (uc). Structures remain largely unaltered in the presence of 0.2 μM ELQ-400 **(C,D)**. Note the presence of mitochrondria (mito) **(B–D)** with a rather electron-dense matrix and cristae (arrows); ld, lipid droplet. Bars in **(A)** = 2.4 μm; **(B)** = 0.25 μm; **(C)** = 1.2 μm; **(D,E)** = 0.40 μm.

**Figure 3:**
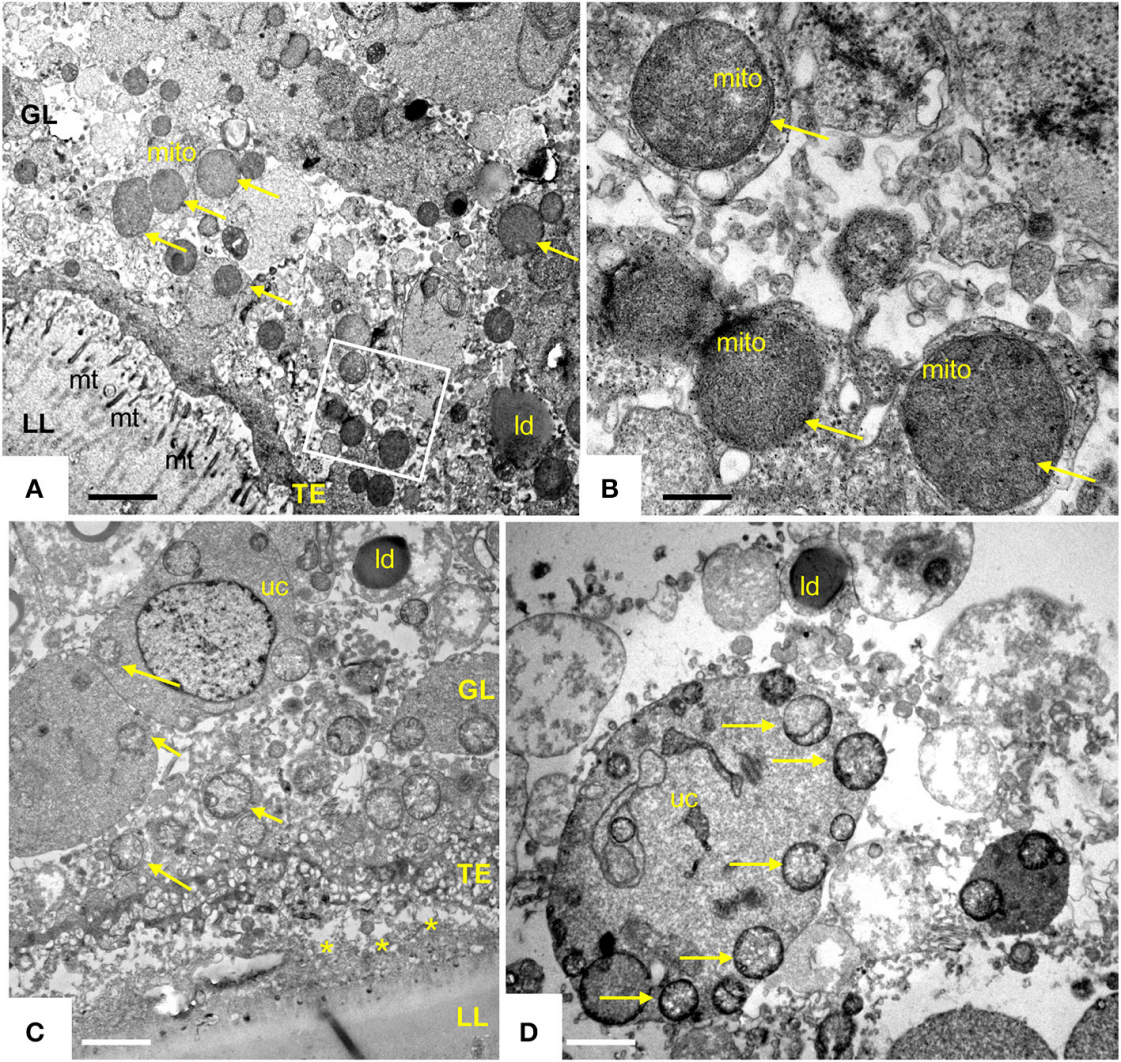
Transmission electron microscopy (TEM) of *E. multilocularis* metacestodes treated with 1 and 2 μM ELQ-400. TEM of metacestode tissue treated with 1 μM **(A,B)** and 2 μM **(C,D)** of ELQ-400 under normoxic (21% O_2_) conditions. The boxed area in **(A)** is shown at higher magnification in **(B)**. Mitochondria (mito) are rounded, progressively less electron dense, on both the GL tissue [**(C)** as well as in undifferentiated cells (uc)] **(D)**, as indicated by the arrows; *indicates separated LL and GL; mt, microtriches; ld, lipid droplet. Bars in **(A)** = 1.2 μm; **(B)** = 0.28 μm; **(C)** = 2.2 μm; **(D)** = 1.8 μm.

Transmission electron microscopy showed that exposure of *E. multilocularis* metacestodes to ELQ-400 under normoxic conditions exerted detrimental effects, apparently in a dose-dependent manner. Metacestodes treated with 0.2 μM ELQ-400 did not show obvious ultrastructural alterations compared to non-treated parasites (Figures 2A,B). A closer look at the mitochondria, which are found within the GL tissue, and which are prominent within the cytoplasm of undifferentiated cells (Figure 2B), revealed that they exhibited different shapes and sizes. Mitochondria were filled with a relatively electron-dense matrix, and contained fine-structured cristae, which were not very prominent but nevertheless clearly discernible. In some metacestodes exposed to 0.2 μM ELQ-400, lipid droplets had formed (Figure 2C). However, this changed upon treatments with 1 μM ELQ-400 (Figures 3A,B), as the overall structural organization of the parasites, especially of the GL, was partially compromised, and mitochondria appeared rounded. Microtriches were still visible being embedded in the LL after 1 μM ELQ-400 treatments (Figure 3A) but not evident anymore after 2 μM treatments (Figure 3C). In addition, the progressive structural damage lead to partial separation of the GL and the LL (Figure 3C), and the presence of rounded mitochondria lacking an electron dense matrix and cristae, which was especially evident in undifferentiated cells (Figure 3D).

### ELQ-400 inhibits the mitochondrial *bc*_1_ complex

**Figure 4:**
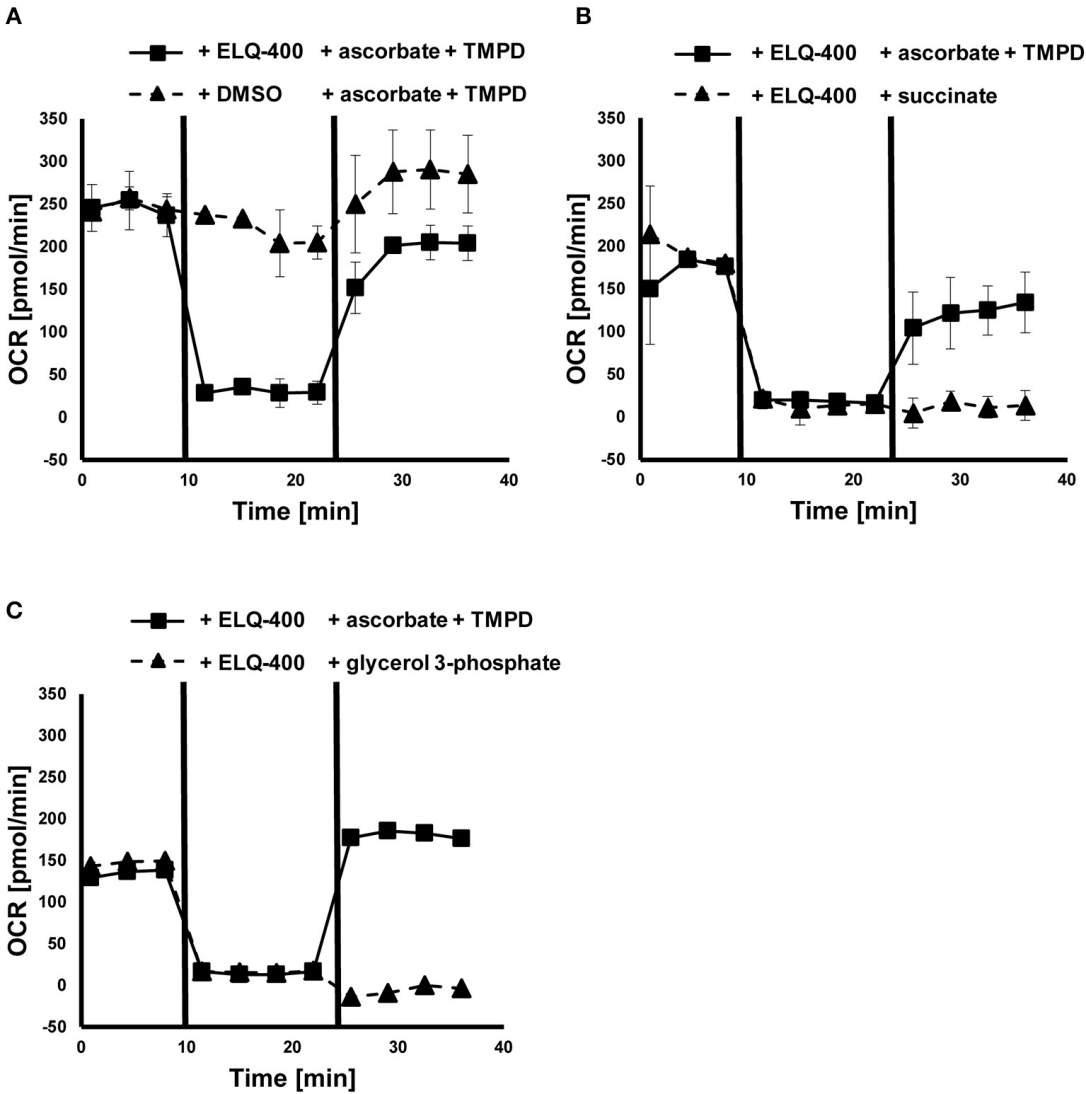
Effects of ELQ-400 on the mitochondrial respiration of *E. multilocularis* germinal layer (GL) cells. The oxygen consumption rate (OCR) of *E. multilocularis* GL cells was measured by Seahorse XFp Extracellular Flux Analyzer. Succinate was given as an initial electron donor. After addition of 1 μM ELQ-400 (but not DMSO), the OCR dropped, but recovered after addition of 20 mM ascorbate and 0.6 mM TMPD, which donate electrons to the mitochondrial respiration chain complex IV, thus downstream of the *bc*_1_ complex **(A)**. OCR dropped after ELQ-400 treatment, but did not recover upon addition of 10 mM succinate **(B)**, nor 10 mM glycerol-3-phosphate (G3P, **C**), both electron-donors which donate electrons upstream of the *bc*_1_ complex. The experiment was repeated two times independently, and one representative plot is shown. Measurements were performed in triplicates, and mean values with standard deviations are shown.

Direct effects of ELQ-400 on isolated GL cells were also assessed by measurement of the OCR in these cells (Figure 4). The addition of ELQ-400 (but not of DMSO) led to an immediate reduction of the OCR of GL cells, and feeding of electrons *via* ascorbate and TMPD, which donate electrons downstream of the *bc*_1_ complex, could restore this effect (Figure 4A). The electron donors succinate or glycerol 3-phosphate, which donate electrons upstream of the *bc*_1_ complex, could not restore the ELQ-400 induced OCR drop (Figures 4B,C). Thus, ELQ-400 inhibits the *bc*_1_ complex of the mitochondrial ETC of *E. multilocularis* GL cells.

### Cytochrome *b* sequence alignments

**Figure 5:**
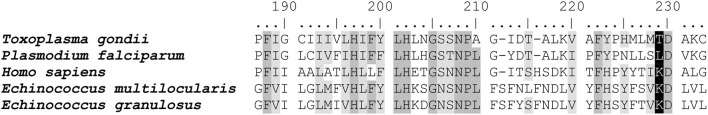
Sequence comparisons of the cytochrome *b* of *Echinococcus* spp. and apicomplexan parasites. Sequence alignment of the Qi site of cytochrome *b* between *E. multilocularis* (C6L2E3), *T. gondii* (S8EQL3) and *P. falciparum* (Q02768), and *H. sapiens* (P00156; EgrG_900000100). The *T. gondii* position 222 is highlighted in black. Conserved sites are marked in gray (four out of five amino acids same) and in light gray (three out of five amino acids same). Position 222 is labeled in black. The entire sequence alignment is given in Supplementary Figure 1.

Cytochrome *b* sequences of the apicomplexan parasites *T. gondii* and *P. falciparum*, as well as *Homo sapiens* were aligned with the sequences of *E. multilocularis* and *E. granulosus* (Supplementary Figure 1). Interestingly, *Echinococcus*, like most eukaryotes, has a lysine at position 229, which was previously shown to reduce Qi site inhibition by ELQs with OCH_3_ at R7 (22) (Figure 5), explaining the lack of activity of these ELQs against *E. multilocularis*.

### Double-treatment of the *bc*_1_ complex and the malate dismutation

**Figure 6:**
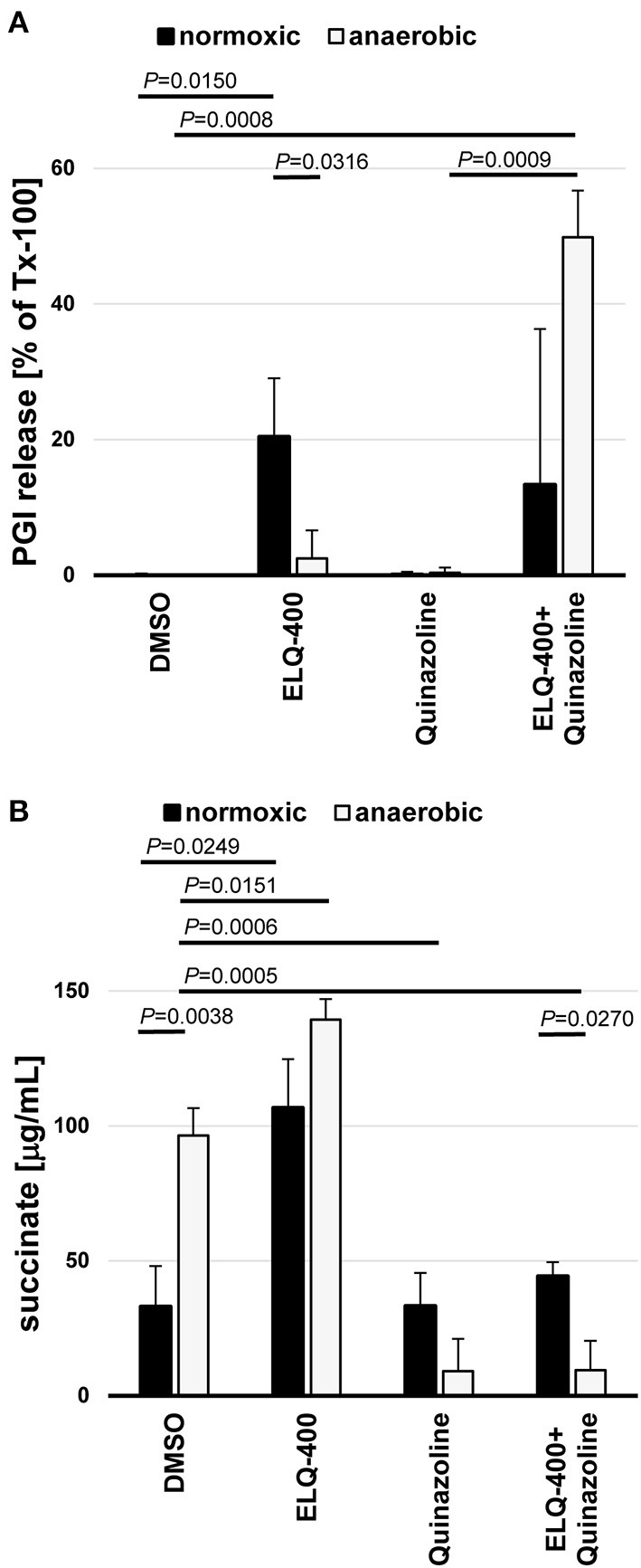
Double-inhibition of the *bc*_1_ complex and the malate dismutation of *E. multilocularis* metacestodes and GL cells. *E. multilocularis* metacestodes were treated with ELQ-400 (10 μM) and or quinazoline (10 μM) for 5 days and PGI release assessed as a quantifiable damage marker in relation to meteacestodes treated with the internal positive control Tx-100 **(A)**. From the same setup, released succinate levels were assessed for each condition by Succinic Acid Assay Kit. Incubations were performed under normoxic (21% O_2_) and anaerobic (0% O_2_) conditions in parallel. DMSO treatment was used a negative control **(B)**. Experiments were performed in biological triplicates, and mean values with standard deviations are given for the relative PGI release **(A)** and the succinate levels **(B)**. *P*-values are given for significant differences with *P* < 0.05.

**Figure 7:**
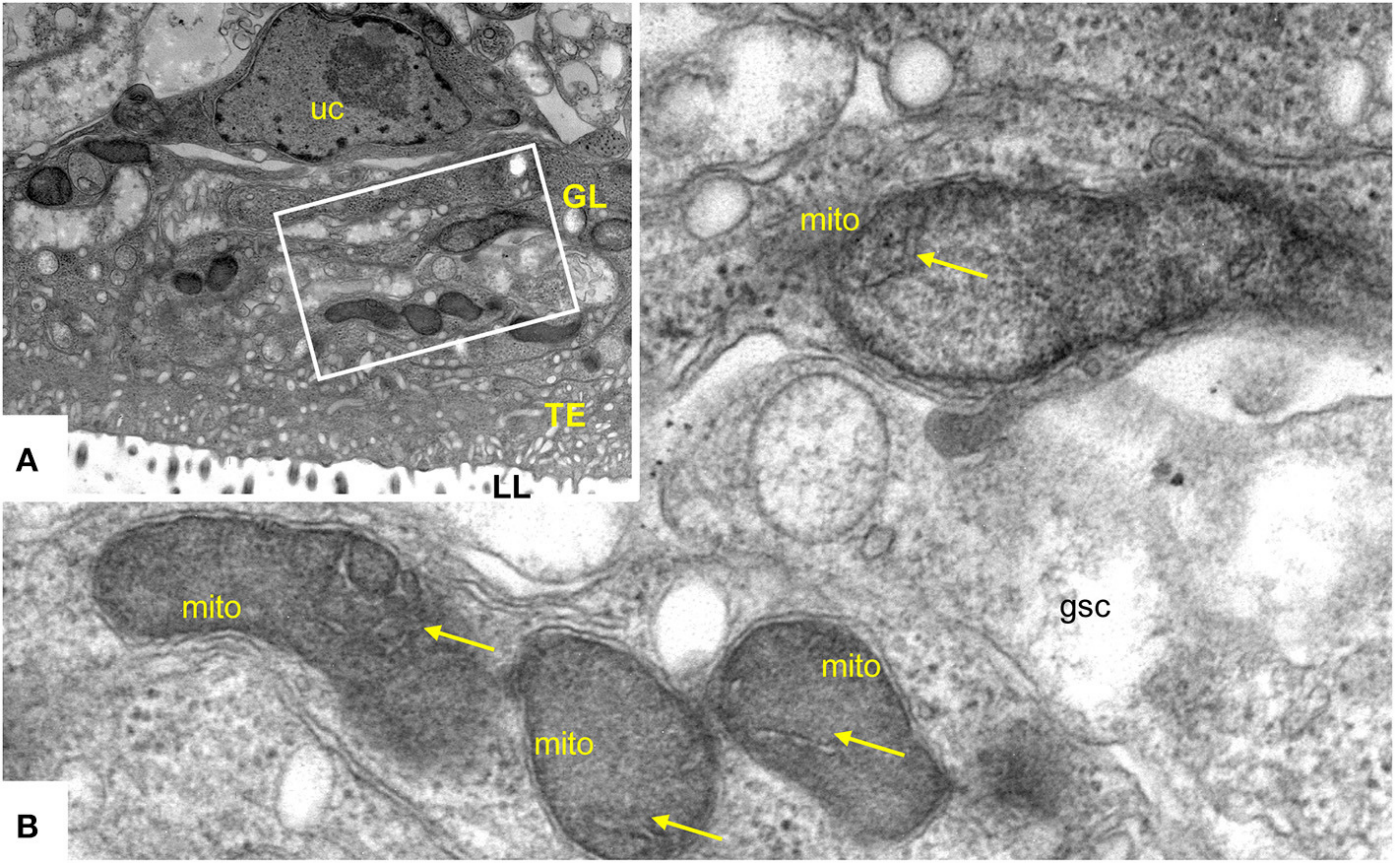
Transmission electron microscopy (TEM) of *E. multilocularis* metacestodes treated with 2 μM ELQ-400 under anaerobic conditions. **(A)** Is a lower magnification view, the boxed area is enlarged in **(B)**. LL, laminated layer; TE, tegument; GL, germinal layer; uc, undifferentiated cell; gsc, glyocogen storage cell; mito, mitochondrion. Arrows point toward mitochondrial cristae which are clearly visible. Bars in **(A)** = 2.4 μm; **(B)** = 0.25 μm.

ELQ-400 was active in the PGI assay when tested against *E. multilocularis* metacestodes at standard normoxic conditions, and its activity was significantly reduced at anaerobic conditions (*P* = 0.0316, Figure 6A). Morphological characterization by TEM confirmed the loss of activity of ELQ-400 under anaerobic conditions: In contrast to the damage exerted by ELQ-400 at 1 and 2 μM under normoxic conditions (Figures 2, 3), metacestodes inspected upon ELQ-400 treatment under anaerobic conditions did not exhibit such alterations, and were largely similar to non-treated metacestodes (Figure 7).

*E. multilocularis* is known to employ also an alternative mitochondrial energy-generating pathway that works oxygen independently, the MD. This pathway leads to the release of succinate as a final electron acceptor, thus succinate is an indicator of MD activity. Succinate levels were significantly increased when metacestodes were incubated under anaerobic conditions (*P* = 0.0038, Figure 6B). ELQ-400 treatment significantly enhanced the release of succinate by metacestodes when compared to the DMSO control (*P* = 0.0249 normoxic, *P* = 0.0151 anaerobic, Figure 6B). Concluding, both anaerobic incubation and ELQ-400 treatment of metacestodes induced the MD.

As a consequence, we then also employed quinazoline the published inhibitor of MD (29), which led to a significant reduction of succinate release under anaerobic conditions (*P* = 0.0006, Figure 6B). Quinazoline treatment alone led only to no PGI release by metacestodes under anaerobic conditions (Figure 6A). The combined treatment of metacestodes with ELQ-400 and quinazoline, thus double inhibition of two mitochondrial pathways ETC and MD simultaneously, led to a significantly increased PGI release, as compared to single treatments under anaerobic conditions (*P* = 0.0009).

## Discussion

This study was investigating novel alternative treatment options against the foodborne disease AE, which is currently treated with benzimidazoles that only act parasitostatic. Other related food-transmitted flatworms that also affect the liver, and are likewise treated by benzimidazoles, are cystic echinococcosis, caused by *E. granulosus*, fasciolosis caused by *Fasciola hepatica* and *F. gigantica*, clonorchiasis caused by *Clonorchis sinensis* or opisthorchiasis caused by *Opisthorchis viverrini* and *O. felineus*. Whereas, AE is of major concern for humans, captive monkeys and dogs, cystic echinococcosis and fasciolosis are additionally also highly relevant diseases in livestock worldwide causing substantial economic losses (9, 10). Thus, novel treatment options against foodborne platyhelminth-infections are needed.

In search for novel treatment options against AE, and possibly other helminth-diseases, we repurposed ELQs that have been shown previously to be highly effective inhibitors of apicomplexan parasites (22). The activities of 13 different ELQs were assessed against metacestodes and isolated GL cells of two isolates of *E. multilocularis*. This small screen revealed that activities of ELQs correlated well between the two parasite isolates, one of which is kept in laboratory mice since the year 1995 (H95) and the other one since at least 2012 (Sval). The data allowed for a small structure activity relationship (SAR) study of the tested ELQs with respect to anti-echinococcal activity. Our data indicates that ELQs with a fluorine at position 5 or 5 and 7 were active, whereas a methoxy group at position 7 led to reduced activity against *Echinococcus*. This is true for the compounds ELQ-121, ELQ-136, ELQ-400, and ELQ-437. The cytochrome *bc*_1_ complex was previously shown to be the target of ELQs (21). The *bc*_1_ complex can be inhibited at two sites that are responsible for building up a proton gradient across the inner mitochondrial membrane: the ubiquinol oxidation site (Qo) or the quinone reduction site (Qi) (22). The Qo site of Apicomplexan *bc*_1_ is inhibited with 5,7-difluoro- or 7 fluoro-position substituents (19), which is in line with the above-mentioned active ELQs against *E. multilocularis*. In contrast, ELQ-271 does not have substituents at positions 5 or 7 and is a Qi site inhibitor (21, 26). The Qi site of the mitochondrial cytochrome *bc*_1_ complex of apicomplexans is also inhibited by compounds with an electron-withdrawing substituent (like a halogen) at position 6, or a 7-methoxy group (19). ELQ-271 was shown to inhibit both human and apicomplexan cytochrome *bc*_1_, whereas ELQs with a 7-methoxy group selectively inhibited the apicomplexan cytochrome *bc*_1_. A better understanding of ELQ selectivity for apicomplexans came from experiments demonstrating that a mutation of the threonine at position 222 of the Qi binding site in *T. gondii* led to resistance to ELQs with a methoxy group at position 7 (22, 37). The *E. multilocularis* cytochrome *b* gene sequence shows a lysine residue at this position, like other eukaryotes, which is well in line with the lack of activity of all ELQs carrying a 7-methoxy group. Within the group of ELQs that are active against *Echinococcus*, this holds true for ELQ-271 and ELQ-400. Thus, Qi inhibitors that are not selective for apicomplexans may be active against *E. multilocularis*. Sequence comparisons with *E. granulosu*s suggest that these Qi inhibitors might also be active against this related parasite. However, experimental testing of ELQs against *E. granulosus* and other platyhelminths infecting the liver has yet to be performed.

Past studies in apicomplexan parasites showed that buparvaquone (alike atovaquone) is a Qo site inhibitor, whereas ELQ-400 is primarily a Qo site inhibitor, but may inhibit both the Qo and the Qi site (38).This might also be the case in *E. multilocularis*, but cannot be further confirmed, as mutagenesis studies cannot be performed to date. Simultaneous treatment of the Qo and the Qi site was shown to be a treatment strategy against *Babesia microti*, and *Plasmodium* infections (26, 39). Our studies demonstrate that the Qi site inhibitor, ELQ-271, also is a potent inhibitor of *E. multilocularis* and had a greater therapeutic index than ELQ-400.

Electron microscopy showed that exposure of *E. multilocularis* metacestodes to ELQ-400 under normoxic conditions exerts detrimental effects in a dose-dependent manner, which closely aligns to the results of the PGI measurements. While 0.2 μM ELQ-400 treatment did not lead to clear-cut changes in the parasite ultrastructure, higher concentrations clearly affected the overall structural organization of the metacestode tissue. In parallel, the mitochondria rounded up, lost their characteristic electron dense matrix, and cristae were dramatically reduced or absent after ELQ-400 treatment. Basically, the observed effects mirrored those seen earlier with the hydroxynaphthoquinone buparvaquone, another cytochrome *bc*_1_ inhibitor (17). However, buparvaquone affected the parasite ultrastructure already at 0.3 μM, by impacting the electron dense mitochondrial matrix.

Our further experiments clearly showed that ELQ-400 affects the mitochondria of *E. multilocularis*, and that it inhibits the *bc*_1_ complex of the mitochondrial ETC. Its activity was depending on the availability of oxygen. Importantly, in the liver of naturally infected hosts, *E. multilocularis* metacestodes do not grow at normoxic conditions, but encounter rather microaerobic conditions (40). This prompted us to include additional experiments targeting a second energy-generating mitochondrial pathway, which functions independent of oxygen: the MD. Even though present in all helminths, and not in mammalian hosts, this pathway has been only little explored for future anthelminthic treatment options. Most of the past studies focused on the free-living nematode *Caenorhabditis elegans* (41, 42) or on parasitic nematodes like *Ascaris suum* and the inhibition of the MD (complex II) by harzianopyridone and atpenin a5 (43) or flutolanil and derivatives (44, 45), or *A. suum* and *Haemonchus contortus* by nafuredin (46). The MD of *E. multilocularis* protoscoleces was described previously, and inhibition of the MD in mitochondria of protoscoleces was achieved by quinazoline and derivatives (29). Furthermore, several benzimidazoles were suggested to interfere with MD of different cestodes (47–50).

We here report for the first time on the direct measurements of MD activity in metacestodes of *E. multilocularis* by measuring succinate, the end product of MD, in this parasite. Succinate production was induced upon anaerobic incubation. ELQ-400 treatment of metacestodes induced succinate production, which could be an indication of an increased MD activity when the *bc*_1_ complex of the ETC is blocked. MD inhibition by quinazoline led to a reduction of succinate production by *E. multilocularis* metacestodes under anaerobic conditions, but did not affect the viability of the parasite. Only a combined treatment approach of the MD inhibitor quinazoline together with the *bc*_1_ inhibitor ELQ-400 led to a strong damage to metacestodes also under anaerobic conditions.

Concluding, this study provides the first proof of concept of a dual inhibition of the mitochondria of *E. multilocularis* metacestodes as a promising approach for future treatment of AE and potentially other helminthiases, in particular the ones caused by platyhelminths. Future studies should aim at the identification of optimized inhibitors that are not toxic to mammalian cells, and exhibit good aqueous solubility and oral bioavailability.

## Data availability statement

The original contributions presented in the study are included in the article/Supplementary material, further inquiries can be directed to the corresponding author.

## Ethics statement

The animal study was reviewed and approved by Amt für Veterinärwesen, Kanton Bern.

## Author contributions

BL-S and JD: conceptualization, project administration, and funding acquisition. SC, RZ, MP, TK, MK, RM, NS, and BL-S: performed experiments with the parasite and extracts. SC and AH performed electron microscopy. JD synthesized the compounds. SC, RZ, MP, AH, JD, and BL-S: performed data analysis. BL-S: writing—original draft preparation and supervision. SC, RZ, MP, TK, MK, RM, NS, AH, JD, and BL-S: contributed in review and editing and approval of manuscript. TK, MP, AH, and BL-S: visualization. All authors contributed to the article and approved the submitted version.

## Funding

This work was supported by a grant from the Novartis Research Foundation to BL-S, the Swiss National Science Foundation (SNSF) grant no. 192072 to BL-S, and VA Merit Review Award BX004522 to JD from the U.S. Department of Veterans Affairs Biomedical Laboratory Research and Development.

## Conflict of interest

The authors declare that the research was conducted in the absence of any commercial or financial relationships that could be construed as a potential conflict of interest.

## Publisher's note

All claims expressed in this article are solely those of the authors and do not necessarily represent those of their affiliated organizations, or those of the publisher, the editors and the reviewers. Any product that may be evaluated in this article, or claim that may be made by its manufacturer, is not guaranteed or endorsed by the publisher.

## Supplementary material

Supplementary Figure 1 Alignment of cytochrome *b* sequences. Sequence alignment of cytochrome b between *E. multilocularis* (C6L2E3), *T. gondii* (S8EQL3), *P. falciparum* (Q02768), and *H. sapiens* (P00156; EgrG_900000100). Conserved sites are marked in gray (four out of five amino acids same) and in light gray (three out of five amino acids same). Dotted rectangles indicate the Qo site, solid lined rectangles the Qi site.
