# Supplementary figures and images for: Dual inhibition of the Echinococcus multilocularis energy metabolism
Source: Front Vet Sci. 2022 Aug 5;9:981664. doi: 10.3389/fvets.2022.981664 (PMC9388906; doi:10.3389/fvets.2022.981664)

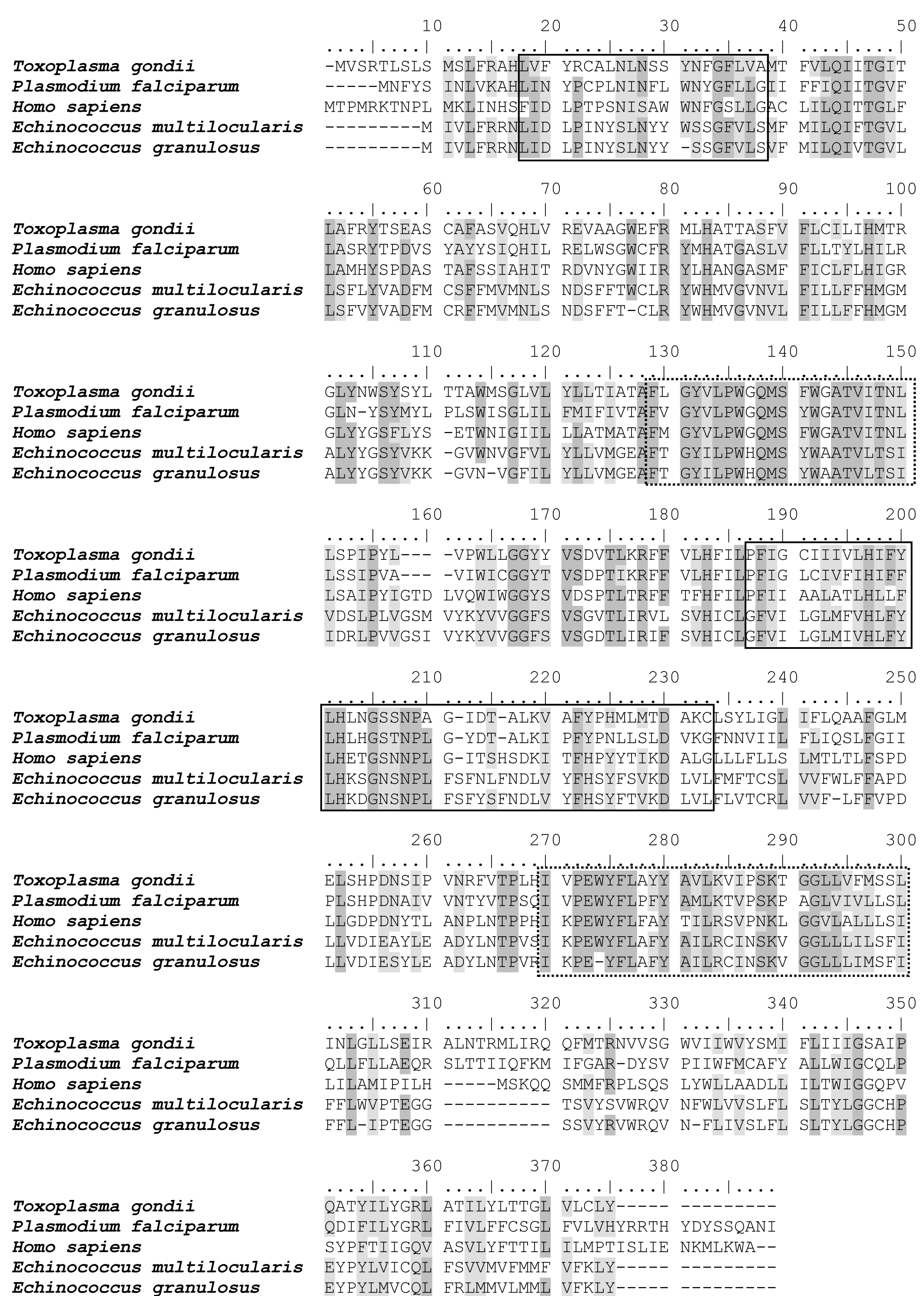

Supplement: Supplementary Figure 1 — Alignment of cytochrome b sequences. Sequence alignment of cytochrome b between E. multilocularis (C6L2E3), T. gondii (S8EQL3), P. falciparum (Q02768), and H. sapiens (P00156; EgrG_900000100). Conserved sites are marked in gray (four out of five amino acids same) and in light gray (three out of five amino acids same). Dotted rectangles indicate the Qo site, solid lined rectangles the Qi site. [file Image_1.TIF]
